# Reframing endometriosis care in low-resource settings: a pragmatic 5R framework for recognition, rule-out, relief, referral and review

**DOI:** 10.3389/frph.2026.1901655

**Published:** 2026-08-17

**Authors:** Sebastian Ken-Amoah, Evans Kofi Agbeno, Samuel Agyeman, Eric Peter Yao Mensah, Betty Anane-Fenin, Godfred O. Boateng

**Affiliations:** 1 Department of Obstetrics and Gynecology, School of Medical Sciences, University of Cape Coast, Cape Coast, Ghana; 2 Department of Obstetrics and Gynecology, Cape Coast Teaching Hospital, Cape Coast, Ghana; 3 Faculty of Health, Dahdaleh Institute for Global Health Research, York University, North York, ON, Canada; 4 Department of Obstetrics and Gynecology, Effia Nkwanta Regional Hospital, Sekondi, Ghana

**Keywords:** care pathway, chronic pelvic pain, diagnostic delay, endometriosis, health systems, infertility, low-resource settings, reproductive health

## Abstract

Endometriosis is a chronic inflammatory gynecological condition associated with severe dysmenorrhea, chronic pelvic pain, deep dyspareunia, cyclic bowel or urinary symptoms and infertility. It is also linked to psychological morbidity, reduced quality of life and lost economic productivity. In low-resource settings, diagnostic and therapeutic delays often reflect pathway failure rather than specialist scarcity alone: symptoms are commonly normalized, first-contact recognition remains weak, differential diagnosis is inconsistent, referral pathways are poorly governed and structured follow-up is frequently absent. This article proposes a pragmatic 5R Framework (Recognition, Rule-Out, Relief, Referral and Review) tailored for symptom-led endometriosis care in low-resource settings. The framework draws on international guidelines, clinical review evidence, African and low- and middle-income country (LMIC) evidence on access barriers, global scoping reviews of policy and service-delivery gaps, imaging guidance, and health-systems evidence on task redistribution. Its central proposition is that endometriosis-compatible symptoms should trigger structured first-contact recognition, safety-oriented rule-out, fertility-sensitive first-line relief, need-based referral and planned review before definitive specialist confirmation is available. The framework is not a validated clinical decision rule; rather, it isa testable pathway model for local adaptation, consensus refinement, facility-level audit and prospective implementation research. Its utility lies in translating established evidence-based principles into an accountable care sequence that permits earlier action without compromising diagnostic caution, reproductive goals or referral responsibilities.

## 1 Introduction

Endometriosis is a chronic inflammatory condition affecting approximately 10% (190 million) of reproductive-aged women and girls globally (1). The condition is associated with severe dysmenorrhea, chronic pelvic pain, deep dyspareunia, bowel and urinary dysfunction, fatigue, and infertility. These physical manifestations frequently drive psychological morbidity, reduced economic productivity, and diminished quality of life (2–5). Although symptoms typically begin during adolescence, they are commonly normalized, undertreated or managed as isolated episodes, particularly where menstrual health literacy, adolescent-friendly care and continuity of care are weak (6, 7). For many patients in low-resource settings, this systemic gap results in a prolonged diagnostic and therapeutic journey, during which clinically meaningful symptoms fail to trigger structured assessment, first-line management or timely specialist referral.

In this paper, low-resource settings refer to health systems or service environments where timely reproductive health care is constrained by limited workforce capacity, uneven diagnostic infrastructure, restricted access to specialist gynecologists, high out-of-pocket costs, weak referral systems and poor continuity mechanisms. Although this paper focuses on low- and middle-income countries (LMIC) and African contexts, resource constraints exist along a continuum, and similar barriers may affect under-served populations within high-income settings. Within these contexts, diagnostic delay reflects a wider service-delivery problem shaped by menstrual stigma, low clinical suspicion, fragmented care and an inability to connect repeated presentations into a coherent clinical pathway. Globally, this delay remains persistent, despite evolving clinical guidance (8). In sub-Saharan Africa, historical misconceptions that endometriosis is rare among women of African descent have entrenched under-recognition and stifled research investment (9, 10). Consequently, as seen in Kenyan narratives, pain dismissal, chronic misdiagnosis, prohibitive costs and specialist scarcity compound the physical, emotional, relational and economic harms experienced by patients (11).

Evidence from LMICs remains sparse, geographically uneven and predominantly facility-based. Across Africa, studies demonstrate wide variations in reported prevalence and clinical pathways due to divergent study populations, diagnostic methodologies, and referral patterns (12, 13). This heterogeneity precludes straightforward burden estimation, highlighting an urgent need for practical care pathways that can navigate diagnostic uncertainty. Because most global research is concentrated in high-income settings, its findings lack direct relevance to health systems where specialist imaging, laparoscopy, pathology, second-line hormonal therapies, multidisciplinary pain management and assisted reproduction are either unavailable or unaffordable (9, 10).

The sparse evidence base from low- and middle-income countries should prompt cautious service adaptation, rather than delayed service improvement. Contemporary international guidance facilitates this approach; both the European Society of Human Reproduction and Embryology (ESHRE) and the National Institute for Health and Care Excellence (NICE) support clinical assessment, imaging and empirical treatment where appropriate, reserving laparoscopy for specific indications. These include persistent symptoms, treatment failure, diagnostic uncertainty, surgical necessity or negative imaging with ongoing clinical suspicion (14, 15). Recent clinical reviews similarly emphasize non-surgical diagnosis and early intervention to mitigate avoidable delays (2, 5, 16, 17). For instance, transvaginal or transabdominal ultrasound can triage patients by identifying endometriomas, adenomyosis, fibroids and some deep disease, though a normal scan cannot exclude superficial peritoneal lesions. While these paradigm shifts enable earlier intervention within constrained systems, they introduce a critical implementation challenge: how can clinicians deliver timely, safe and reviewable care when specialist confirmation is delayed or entirely unavailable?.

That implementation challenge remains underdeveloped within reproductive health policy and service-delivery guidance. Recent global scoping reviews have identified stark deficiencies in endometriosis policy, delivery systems and region-specific guidelines, particularly in lower-income settings (18, 19). The immediate practical challenge is to ensure that awareness-raising campaigns and specialist capacity-building translate into operational pathways. Such pathways must enable clinical symptoms, fertility goals, treatment responses and referral thresholds to be managed coherently across first-contact, district and specialist tiers. Achieving this requires reframing endometriosis from a disease that is actionable only upon specialist confirmation to a chronic reproductive health condition that is systematically recognized, triaged, treated, referred and reviewed. To operationalize this shift, this paper proposes a pragmatic “5R Framework” (Recognition, Rule-Out, Relief, Referral, and Review) designed to translate contemporary guidance into a tiered, fertility-sensitive, and reviewable care pathway for low-resource settings.

## 2 Derivation and intended use of the 5R framework

The 5R Framework was developed to operationalize the paradigm shift proposed in this paper: that endometriosis care in low-resource settings must not remain contingent on specialist confirmation before meaningful clinical action begins. Instead, clinically significant symptoms should immediately trigger a tiered pathway of recognition, safe assessment, first-line care, referral and review, while definitive diagnosis is pursued as indicated. The framework is therefore intended to systematically organize the safe clinical and administrative actions available to clinicians when specialist imaging, laparoscopy, pathology, fertility services or multidisciplinary care are delayed, unavailable or unaffordable.

The 5R Framework was developed through an expert-informed narrative synthesis rather than a formal systematic or scoping review. The literature informing the framework was identified through iterative searches of major biomedical databases, and guideline repositories, supplemented by citation tracking from key guidelines, clinical reviews, and regionally relevant studies. Priority was given to contemporary international guidance on endometriosis diagnosis and management, recent clinical reviews, African and LMIC literature on access barriers and diagnostic delay, imaging guidance relevant to ultrasound triage, and WHO health-systems literature on task redistribution. Sources were selected for their relevance to the framework's core implementation problem: how to support safe, fertility-sensitive, and reviewable care when specialist confirmation is delayed, unavailable or unaffordable. Because the process was narrative and expert-driven, the framework should be interpreted as a hypothesis-generating pathway model requiring consensus refinement and empirical testing rather than as a guideline derived from systematic evidence grading.

This synthesis identified five overlapping evidence streams that informed the framework:

- Contemporary endometriosis guidance: Recent guidelines and clinical reviews shaped the diagnostic logic of the model, particularly the transition away from mandatory laparoscopic confirmation towards clinical assessment, available imaging and empirical treatment (2, 15–17, 20).
- Epidemiological and delay data: Evidence on the global disease burden and pervasive diagnostic delays reinforced the necessity of early recognition and continuity of care over fragmented, episodic management (1, 8).
- African and LMIC literature: Regional data highlighted the structural barriers that undermine conventional, specialist-dependent pathways, including low public and provider awareness, historical misconceptions regarding endometriosis in African women, prohibitive costs, specialist scarcity, weak referral networks and poor documentation (9–13).
- Gynecological imaging guidance: Advanced imaging literature informed the distinction between using ultrasound for triage and avoiding false reassurance following a normal scan, given that superficial peritoneal disease and some deep lesions may remain undetected. It also highlighted the potential role of structured ultrasound assessment, including International Deep Endometriosis Analysis terminology, in improving the detection of endometriomas, adenomyosis and deep disease when clinicians or sonographers have appropriate training (2, 14, 21, 22)
- Health systems and task-shifting frameworks: WHO health-systems guidance provided the foundational implementation principle that specific clinical functions can be safely distributed across health care cadres when supported by structured training, decision-support tools, explicit referral thresholds, supervision and robust accountability mechanisms (23).

**Figure 1:**
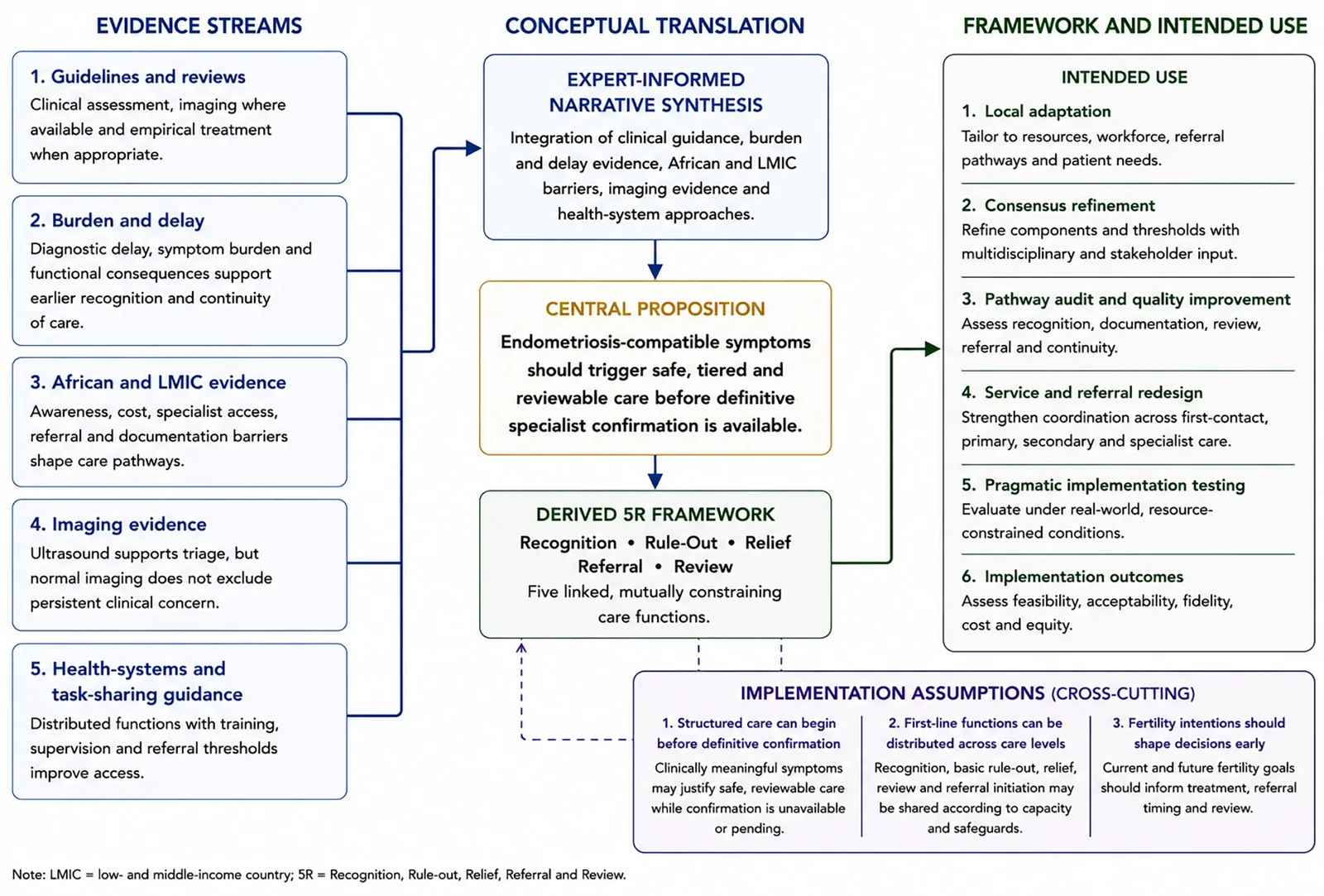
Derivation logic and intended use of the 5R framework. Five evidence streams were integrated through an expert-informed narrative synthesis to generate the central proposition that endometriosis-compatible symptoms should trigger safe, tiered and reviewable care before definitive specialist confirmation is available. From this proposition, the 5R Framework is derived: Recognition, Rule-Out, Relief, Referral and Review. The figure also shows cross-cutting the implementation assumptions and intended applications, including local adaptation, consensus refinement, pathway audit, service and referral redesign, implementation testing and evaluation of feasibility, acceptability, fidelity, cost and equity.

The derivation logic of the 5R Framework is illustrated in Figure 1.

The central proposition is that endometriosis-compatible symptoms should trigger safe, tiered and reviewable care before definitive specialist confirmation is available. The pathway is built around five functions that a safe and reviewable care system should be able to perform. Recognition prevents clinically significant symptoms from being dismissed as normal menstruation. Rule-Out protects against diagnostic closure by requiring consideration of urgent conditions and common mimics. Relief enables appropriate first-line treatment when pregnancy is not desired and immediate referral is not required. Referral links escalation to clinical severity, suspected disease complexity, fertility intention and treatment response. Review converts empirical care into monitored chronic care and generates the documentation needed for accountability.

The sequence of the five functions is deliberate but not rigid. Recognition and Rule-Out are paired because earlier suspicion is unsafe if alternative causes of pelvic pain are ignored. Relief and Referral are paired because empirical treatment needs boundaries, particularly when symptoms are severe, function is impaired, imaging suggests endometrioma or deep disease, or pregnancy is desired. Review closes the loop because pathways in constrained systems often fail after the first visit: patients may receive analgesics, antibiotics or hormonal treatment without systematic reassessment of response, adverse effects, fertility goals or referral completion. The model therefore treats follow-up as a core clinical function rather than an optional administrative task.

The framework rests on three explicit assumptions. First, endometriosis-compatible symptoms can be systematically documented and managed within a structured pathway even where definitive diagnosis is not immediately accessible. This does not require assigning a premature disease label; rather, it requires that clinically significant symptoms trigger assessment, safety checks, appropriate first-line care, referral when indicated and planned review. Second, selected first-line care functions can be safely distributed across health care tiers, provided that referral thresholds, supervision and safeguards are clearly defined. Third, fertility intentions must be assessed early, as prolonged hormonal suppression is inappropriate for individuals actively seeking pregnancy without concurrent fertility evaluation or specialist referral. These assumptions remain testable and may vary by context, which is why the model is presented as a framework for adaptation rather than a rigid universal protocol.

The 5R Framework is not a validated clinical decision rule, a formal guideline or a substitute for specialist care. It was developed without a Delphi consensus, formal co-design, structured stakeholder consultation or empirical testing. In particular, patients, adolescents and people with lived experience did not participate in the development of the current model. This is a key limitation: the framework reflects an expert-informed synthesis rather than a stakeholder-derived pathway, and its acceptability, feasibility, cultural fit and contextual validity should not be assumed. Formal co-design and structured consultation with patients, adolescents, community representatives, frontline clinicians, sonographers, fertility-care providers, gynecologists and health-system managers are therefore necessary before local implementation or claims of contextual validity. The current purpose of the framework is to articulate a coherent, testable paradigm for care where specialist imaging, laparoscopy, pathology, fertility services or multidisciplinary care are delayed, unavailable, or unaffordable.

## 3 The 5R framework

**Figure 2:**
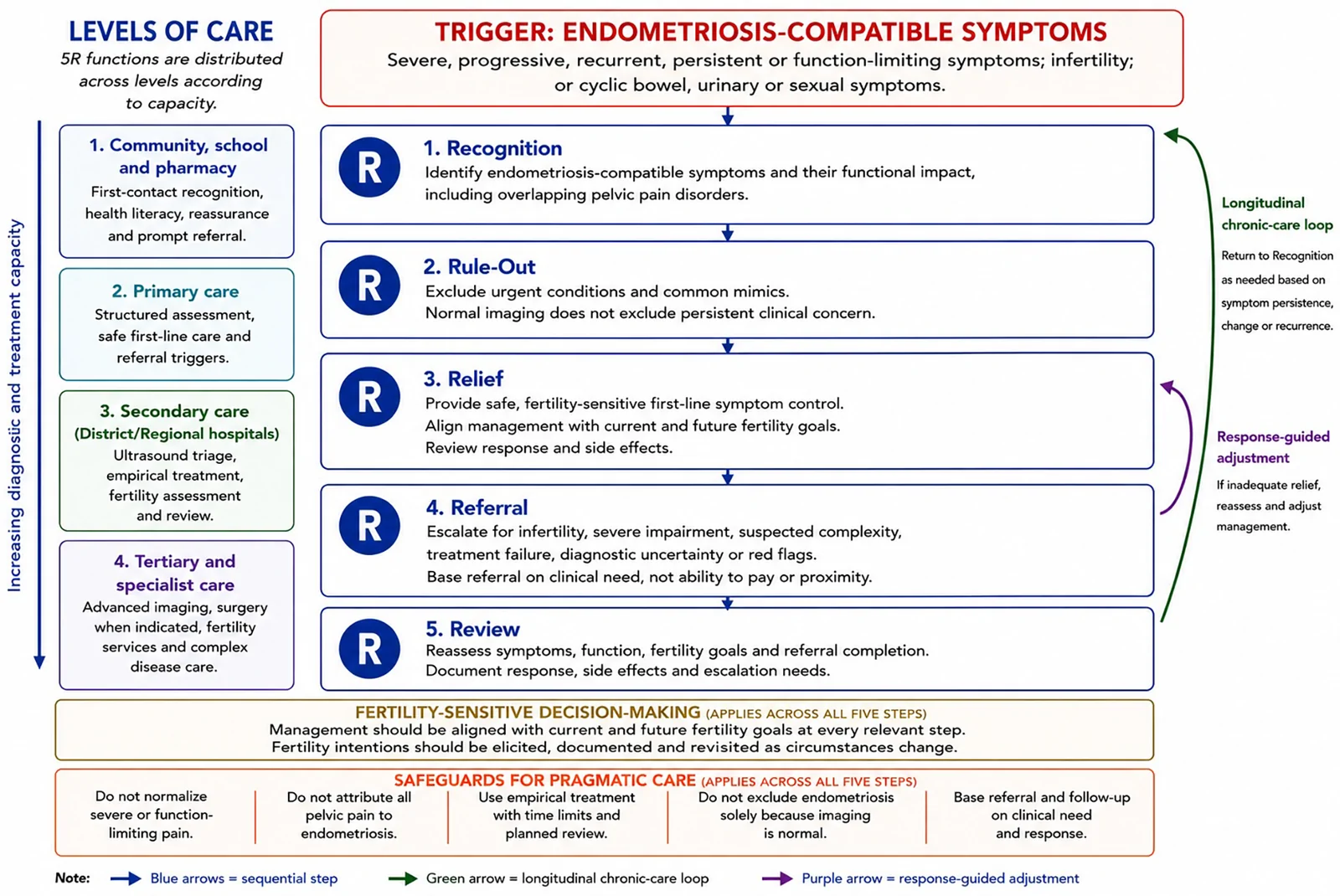
Operational pathway of the 5R framework across levels of care. The5R framework organizes care for endometriosis-compatible symptoms care into five linked functions: Recognition, Rule-Out, Relief, Referral and Review. The pathway is distributed across levels of care according to diagnostic and treatment capacity, with a longitudinal chronic-care loop, response-guided adjustment and fertility-sensitive decision-making applying across the care process. Safeguards caution against normalizing severe or function-limiting pain, attributing all pelvic pain to endometriosis, continuing empirical treatment without time limits and planned review, excluding endometriosis solely because imaging is normal, and basing referral or follow-up on anything other than clinical need and response.

The 5R Framework operationalizes the paradigm shift proposed in this paper by treating endometriosis-compatible symptoms as a clinical trigger for structured care before specialist confirmation is achieved. It is explicitly designed for health care environments where a definitive diagnosis or specialist services are delayed, unavailable or unaffordable. The framework is not intended to label all patients with dysmenorrhea or chronic pelvic pain as having endometriosis. Rather, it uses symptoms compatible with endometriosis and overlapping pelvic pain disorders as triggers for structured assessment, safe exclusion of urgent conditions and common mimics, first-line symptom relief, need-based referral, and planned review. While the five core functions are presented sequentially for clarity, the clinical pathway is inherently iterative: symptoms evolve, reproductive goals change, treatment responses clarify and referral thresholds may be met over time. The overall pathway structure and cross-cutting safeguards of the 5R Framework are shown in Figure 2.

Recognition serves as the critical entry point. Clinicians and first-contact providers should identify endometriosis-compatible symptoms when dysmenorrhea is severe, progressive, recurrent, function-limiting or poorly responsive to appropriately used first-line analgesia. Suspicion should be heightened when this pain is chronic and accompanied by deep dyspareunia, dyschezia, cyclic bowel or urinary symptoms, fatigue, infertility, or repeated health care seeking. First-contact providers do not need to establish a definitive diagnosis; their role is to recognizewhen menstrual pain warrants structured assessment and follow-up. For implementation purposes, severe or function-limiting pain may be operationalized locally using pragmatic criteria such as pain causing school or work absenteeism, inability to perform usual activities, repeated unscheduled care-seeking, escalating analgesic use, or poor response to appropriately used first-line analgesia. Persistent symptoms may be defined as ongoing clinically significant pain or functional limitation after a time-bound therapeutic trial, commonly within approximately three months, although earlier escalation is warranted when red flags, infertility, suspected endometrioma, deep disease, bowel or urinary symptoms, or severe impairment are present.

Rule-Out ensures that pragmatic, empirical care remains safe. Endometriosis must not become an uncritical default explanation for pelvic pain, as pregnancy-related complications, acute pelvic inflammatory disease, sexually transmitted infections, ovarian cyst accidents, adnexal masses, fibroids, adenomyosis, urinary tract diseases, gastrointestinal pathologies and malignancies can present with overlapping symptoms. The presence of clinical instability, fever, acute abdominal signs, abnormal bleeding, a palpable pelvic mass, unexplained weight loss, hematuria, rectal bleeding or severe new-onset pain must prompt immediate escalation rather than routine empirical management.

Relief provides structured, first-line symptom control for patients who do not require urgent escalation and are not actively seeking conception. Therapeutic options include non-steroidal anti-inflammatory drugs (NSAIDs) where clinically appropriate, hormonal suppression, counseling and supportive measures. Choice should be guided by contraindications, resource availability, affordability, side-effect profiles, patient preference and reproductive intent (2, 14). Fertility intentions must be documented prior to initiating hormonal suppression, as an active desire for pregnancy fundamentally alters the management and referral pathway. Notably, while symptom relief may support a clinical suspicion of endometriosis, it neither confirms nor excludes the diagnosis.

Referral is driven by clinical necessity rather than diagnostic certainty alone. Prompt escalation is indicated for patients experiencing infertility or desiring immediate conception, those with suspected endometrioma or deep disease, individuals with persistent symptoms despite structured first-line care, or cases involving severe functional limitation, significant bowel or urinary symptoms, a pelvic mass, diagnostic uncertainty or features suggesting serious alternative pathology. Suspected endometriosis must be integrated into standard infertility pathways, given that the prevalence of laparoscopically confirmed disease is high among women undergoing evaluation for unexplained infertility (24).

Review converts empirical interventions into accountable chronic care. Systematic follow-up must reassess pain trajectories, functional status, adverse treatment effects, fertility intentions, referral completion, available imaging findings and the need for further escalation. A scheduled review within approximately three months is reasonable for most patients initiating first-line therapy, though exact timing must adapt to symptom severity, patient age, treatment tolerability, red flags, reproductive goals and local service capacity.

Adolescents require additional cross-cutting ethical safeguards across all five functions. Assessment and treatment should follow applicable local legal and policy requirements for consent, assent, confidentiality and parental or guardian involvement, while recognizing adolescents' evolving capacity and participation in decisions about their care (25). Where hormonal treatment is considered, clinicians should provide age-appropriate counseling on expected benefits, adverse effects, alternatives and the need for review. Fertility-related discussions should be developmentally appropriate and should address present and future reproductive goals, menstrual health and symptom control without assuming immediate fertility intentions. These safeguards are particularly important where menstrual stigma, power asymmetries and limited adolescent-friendly services may restrict disclosure of pain, sexual symptoms or treatment concerns.

The five components are mutually constraining. Recognition without Rule-Out risks diagnostic closure and overdiagnosis. Rule-Out without Relief leaves patients navigating protracted investigations without symptom control. Relief without Referral risks delaying essential care for infertility, endometriomas, deep disease or refractory pain. Referral without local Review abandons patients who face geographic, financial or waiting-list barriers. Finally, Review without robust documentation fails to render the disease burden visible to facilities and health systems. The framework is therefore best understood as a safety architecture for pragmatic care rather than a shortcut around diagnostic reasoning. The core components, minimum actions and integrated safeguards of the 5R Framework are summarized in Table 1.

**Table 1:** Core components, minimum actions and safeguards of the 5R framework for endometriosis care in low-resource settings.

Component	Core question	Minimum action	Safeguard
Recognition	Are the symptoms compatible with endometriosis or a related pelvic pain disorder?	Screen for severe dysmenorrhea, chronic pelvic pain, deep dyspareunia, dyschezia, cyclic urinary or bowel symptoms, infertility and functional impairment.	Do not normalize severe, progressive or function-limiting menstrual pain.
Rule-Out	Is there an urgent condition or common mimic?	Evaluate pregnancy status, infection risk, acute abdominal signs, pelvic masses, urinary or gastrointestinal causes and malignancy red flags.	Avoid diagnostic closure; do not treat all pelvic pain as endometriosis.
Relief	Can safe first-line symptom control begin now?	Initiate NSAIDs where appropriate, offer hormonal suppression if pregnancy is not desired, provide counseling and schedule a planned review.	Do not initiate or continue hormonal suppression without documenting fertility intentions and reviewing treatment response.
Referral	Is specialist escalation indicated?	Refer promptly for infertility, active desire for conception, suspected endometrioma or deep disease, severe limitation, treatment failure or diagnostic uncertainty.	Do not overlook active fertility desires, palpable masses or significant bowel and urinary dysfunction.
Review	Is care delivering therapeutic benefit and continuity?	Reassess pain trajectories, functional status, adverse effects, fertility goals, referral completion and health outcomes.	Do not allow endometriosis-compatible symptomsto remain undocumented, unreviewed or unmonitored within the health tier.

NSAIDs, nonsteroidal anti-inflammatory drugs.

## 4 From framework to implementation and testing

### 4.1 Implementing the minimum package

The 5R Framework can be operationalized as a minimum service package across levels ofcare, with each tier contributing to Recognition, Rule-Out, Relief, Referral and Review according to its capacity. At first contact, the priorities aremenstrual health literacy, recognition of clinically significant pain and assessments. At primary-care and outpatient levels, priorities shift to structured symptom documentation, fertility-intention assessment, red-flag identification, safe first-line care and a clear plan for review or referral.

Intermediate facilities, including district and regional hospitals, can incorporate pelvic examinations where appropriate, pregnancy testing, targeted infection assessments, ultrasound triage where available, basic infertility evaluations and referral coordination. Finally, tertiary and specialist services provide advanced imaging, complex surgery, multidisciplinary pain care and assisted reproduction, while systematically feeding clinical data back to referring facilities to ensure local continuity of care. This tiered approach preserves specialist resources for complex disease while preventing first-contact services from becoming passive holding points for patients awaiting surgical confirmation. A proposed minimum implementation package by level of care is outlined in Table 2.

**Table 2:** Minimum implementation package for the 5R framework by level of care.

Level of care	Core responsibility	Minimum actions	Escalation point
Community, school, pharmacy and non-specialist first contact	Identify clinically significant menstrual pain and facilitate early clinical assessment.	Screen for severe recurrent pain, school/work absenteeism, escalating analgesic use and persistent symptoms.	Severe, progressive or function-limiting pain; infertility; cyclic bowel, urinary or sexual symptoms.
Primary care and outpatient services	Assess symptom trajectories, exclude urgent conditions and initiate safe first-line management.	Take a structured history of pain severity, cyclicity, functional limitation, dyspareunia, bowel or urinary symptoms and prior treatments; assess pregnancy possibility and infection risk; document fertility intentions; initiate appropriate analgesia or hormonal suppression where pregnancy is not desired; provide counseling; and schedule review.	Presence of red flags, pelvic mass, infertility, immediate desire for conception, treatment failure or clinical uncertainty.
Intermediate facilities(District and regional hospitals)	Provide structured clinical evaluation, ultrasound triage, empirical care and referral coordination.	Perform pelvic examinations where appropriate, pregnancy testing, targeted infection screening, ultrasound, counseling and scheduled reviews.	Suspected endometrioma, deep disease, significant bowel or urinary dysfunction, refractory pain or requirement for surgery.
Tertiary and specialist services	Manage complex, refractory, fertility-related or surgically indicated endometriosis.	Deliver advanced imaging, surgical assessment, laparoscopic management where indicated, fertility interventions and feedback loops to referring facilities.	Recurrent or complex disease, advanced assisted reproduction requirements or persistent symptoms despite specialist input.

Adenomyosis is an important overlapping condition within this pathway. It may present with dysmenorrhea, chronic pelvic pain, abnormal uterine bleeding and subfertility, and may be difficult to distinguish from endometriosis at first contact, particularly where ultrasound expertise is limited. The early 5R functions therefore apply to endometriosis-compatible symptoms and related pelvic pain disorders, including adenomyosis, while referral and imaging help refine diagnosis and management over time. Training curricula and decision-support tools should be organized around the five pathway functions rather than specialist disease knowledge alone. Practical training modules should equip frontline cadres to evaluate pain severity, cyclicity, functional impairment, deep dyspareunia, dyschezia, cyclic urinary or bowel symptoms, previous treatment for pelvic inflammatory disease or urinary tract infections, analgesic use and fertility intention. Advanced ultrasound training should be considered a central implementation strategy where equipment is available. Training clinicians and sonographers to identifyendometriomas, adenomyosis, adhesions, sliding sign abnormalities and features suggestive of deep disease may improve triage, reduce unnecessary diagnostic laparoscopy and strengthen referral prioritization. Structured approaches, such as the International Deep Endometriosis Analysis consensus provide a practical basis for standardizing sonographic evaluation, while diagnostic accuracy studies support the role of transvaginal ultrasound in identifying deep endometriosis (21, 22). However, a normal scan should not override persistent endometriosis-compatible symptoms, functional impairment, infertility, or poor response to structured first-line care. Training must also define the boundaries of safe local management, because task-shifting is appropriate only when robust supervision, explicit referral thresholds and clear accountability mechanisms are institutionalized (23).

Policy and service design should embed endometriosis-compatible symptoms into existing adolescent health, menstrual health, chronic pelvic pain, infertility and primary-care platforms. Recent global scoping reviews demonstrate that endometriosis policy, delivery systems and region-specific care guidance remain fragmented, with profound gaps in many lower-resource settings (18, 19). The 5R Framework offers a practical mechanism for translating policy recognition into actionable symptom triggers, first-line pharmaceutical access, standardized referral criteria, documentation fields, and follow-up indicators.

### 4.2 Safeguards, indicators and accountability

Safeguards are foundational to implementation because empirical treatment improves access but introduces clinical risk if it replaces differential diagnosis, fertility assessments or structured follow-up. First-line care must therefore be strictly linked to a planned review, referral criteria must remain explicit, and escalation should be dictated by clinical need rather than patient persistence, financial means or proximity to tertiary centers. Review is not a passive follow-up encounter; it is the mechanism by which the pathway evaluates whether the initial clinical judgment remains valid. At review, clinicians must document symptom trajectories, adverse effects, fertility goals, referral status, new-onset red flags and reasons for escalation or continued local management. This is particularly vital where normal imaging, temporary symptom relief or geographic distance from specialist services might generate false reassurance.

These safeguards should be embedded within health registers, referral forms, patient-held charts or electronic medical records rather than left to individual clinical memory. Accountability should be assigned to an identifiable service structure rather than left solely to individual clinician discretion. Each implementing facility should identify the unit or role responsible for pathway oversight, such as a reproductive health coordinator, gynecology unit, adolescent health focal person, infertility clinic, quality-improvement committee or district health management team. These structures should periodically review whether key pathway steps are being completed, including documentation of fertility intentions before hormonal suppression, review attendance after first-line treatment, referral completion and escalation of red-flag presentations. Facilities may operationalize this through outpatient registers, referral logs, patient-held records, electronic medical records or quarterly audit meetings. The framework is therefore accountable only when documentation is linked to named responsibility, periodic review and corrective action. Service-facing indicators are essential to measure whether the pathway is functioning, rather than whether every patient has obtained definitive surgical confirmation. Useful implementation metrics should capture:

- The proportion of patients presenting with severe dysmenorrhea or chronic pelvic pain screened for endometriosis-related symptoms;
- The proportion of patients with reproductive intentions explicitly documented;
- The proportion initiated on review-linked first-line treatment;
- The proportion reviewed within the recommended three-month window following treatment initiation;
- Referral completion rates and documented treatment responses at follow-up

Key implementation risks and mitigation mechanisms are summarized in Table 3.

**Table 3:** Implementation risks and mitigation mechanisms for the 5R framework.

Risk	Potential harm	Mitigation mechanism
Overdiagnosis of endometriosis	Missed pregnancy complications, pelvic infections, adnexal masses, gastrointestinal or urinary diseases and malignancy red flags.	Apply Rule-Out criteria before empirical treatment or routine specialist referral.
False reassurance from normal imaging	Delayed reassessment, diagnostic closure and prolonged patient suffering.	Interpret imaging findings in relation to symptoms, reproductive intent, treatment response and functional impairment.
Indefinite empirical treatment	Delayed specialist referral, postponed fertility evaluations and unmanaged symptom progression.	Mandate time-bound therapeutic trials linked to structured review intervals and explicit escalation criteria.
Failure to assess reproductive goals	Inappropriate or prolonged hormonal suppression despite an active desire for pregnancy.	Document fertility intentions before prescribing hormonal therapies and fast-track a referral pathway when conception is sought.
Referral inequity	Structural exclusion of impoverished, rural, adolescent or marginalized patients from specialist networks.	Establish explicit clinical-need criteria, implement referral tracking and prioritize referrals based on clinical severity and reproductive goals.
Suboptimal documentation	Fragmented continuity of care, unquantified disease burden and weakened systemic accountability.	Systematically record suspected and confirmed disease, treatment response, referral milestones and follow-up metrics.

### 4.3 Testing the framework

The 5R Framework generates clear, testable propositions for future implementation research. We hypothesize that, compared with unstructured episodic care, locally adapted implementation of the 5R Framework will improve early recognition, documentation of fertility intentions, review completion and clinically appropriate referral without increasing diagnostic closure or indefinite empirical treatment. A locally adapted pathway would also be expected to reduce repeated empirical treatment without reassessment, including inappropriate antibiotic use and generate more robust routine data on suspected as well as confirmed disease. These propositions allow the framework to be rejected, refined or adapted if empirical testing reveals poor follow-up, inappropriate treatment, referral bottlenecks or inequitable access.

Testing should proceed in distinct methodological stages. First, consensus methods should be used to refine the framework's minimum functions and safeguards rather than to impose a single universal protocol. A Delphi process may help define broad domains such as core symptom triggers, minimum documentation fields, fertility safeguards, referral principles and review expectations. However, country-specific or region-specific adaptation remains essential because treatment availability, ultrasound capacity, referral geography, financing and workforce composition vary substantially across settings. Second, facility-level pathway audits can identify baseline gaps in care. Third, pragmatic pilots, before-and-after implementation studies and mixed-methods evaluations can assess whether the framework is feasible, acceptable, adopted with fidelity and associated with improved continuity processes. Finally, rigorous cost and equity analyses should determine whether the pathway improves access without shifting financial or navigational burdens onto vulnerable patients least able to absorb them.

Evaluation metrics should encompass implementation, process and patient-centered outcomes. Implementation outcomes include feasibility, acceptability, adoption, fidelity, referral functionality, cost and equity. Process outcomes should measure completion of symptom assessment, documentation of fertility intentions, initiation of review-linked care, referral appropriateness and review attendance. Finally, patient-centered outcomes must capture pain trajectories, functional status, school or work participation, treatment satisfaction, fertility pathway progression and the perceived clinical validation of symptoms.

## 5 Discussion

The 5R Framework should be interpreted as a hypothesis and implementation model rather than a completed evidence synthesis or formal clinical guideline. Its primary contribution is to translate existing endometriosis guidance into operational pathway logic for settings where diagnosis and care are distributed across non-specialist first-contact interfaces, primary care, intermediates facilities, infertility networks and tertiary centers. This translation is critical because contemporary guidance no longer positions laparoscopy and histology as the universal entry points to care. ESHRE's 2022 guideline explicitly challenges the status of laparoscopy and histology as sole gold-standard diagnostic modalities, while NICE provides structured guidance on referral, diagnosis, treatment and care when fertility is a priority (14, 15).

The framework's distinct strength lies in making this paradigm shift operational for low-resource settings. It does not assume that every clinical environment can offer specialist imaging, laparoscopy, multidisciplinary pain management or advanced fertility services at first presentation. Instead, it defines the essential functions a safe care pathway must perform when definitive confirmation is delayed: recognizing clinically meaningful symptoms, ruling out urgent conditions and common clinical mimics, offering appropriate first-line relief, referring according to clinical need and systematically reviewing treatment response. Consequently, the framework serves as a practical bridge between international guideline principles and constrained reproductive health systems.

This reframing has implications beyond diagnostic delay because it positions endometriosis within integrated reproductive health systems rather than at the margins of specialist gynecology. Severe dysmenorrhea, chronic pelvic pain, deep dyspareunia, cyclic bowel or urinary symptoms and infertility are frequently encountered through disparate entry points, yet they often represent overlapping presentations of the same underlying pathology. A unified pathway approach renders these diverse presentations visible to each other, reducing the risk that adolescents, chronic pain patients and individuals seeking fertility care navigate parallel, siloed services without continuity. In low-resource settings, the central challenge is therefore not simply a missing technology but the absence of a reliable clinical sequence linking recognition, safe first-line management, fertility-sensitive escalation and structured review.

The 5R Framework should therefore be conceptualized as an adaptation tool rather than an alternative to ESHRE, NICE or specialist care. International guidance establishes the principles of diagnosis and management; the 5R Framework organizes those principles across health systems where ultrasound expertise, laparoscopy, pathology, specialist gynecologists and fertility services are unevenly distributed. It does not lower the threshold for diagnostic caution. As outlined in Tables 1, 2, the framework links minimum clinical actions to safeguards and allocates implementation responsibilities across levels of care. A low-resource pathway can fail in two directions: by delaying care until specialist confirmation is accessible, or by normalizing unstructured empirical treatment without reassessment, referral or fertility-sensitive decision-making.

The framework has notable limitations. It has not been empirically validated, and evidence forits components varies in strength. Guidance on non-surgical diagnosis, imaging-informed assessment and empirical treatment is comparatively robust, whereas evidence on task-shifting, referral governance, low-resource ultrasound triage, financing and long-term follow-up remains sparse. The framework was also developed without formal co-design or structured stakeholder consultation; it is therefore an expert-informed synthesis rather than a stakeholder-derived pathway, and its contextual acceptability should not be assumed. These limitations require co-design and empirical testing before implementation or claims of contextual validity. Task-shifting should likewise be interpreted as a health-systems implementation principle rather than endometriosis-specific evidence. These limitations require co-design and empirical testing before implementation or claims of contextual validity.

Next steps should combine formal co-design, context-specific adaptation and empirical validation. Before implementation, patients, adolescents, people with lived experience, community representatives, frontline clinicians, sonographers, fertility-care providers, gynecologists and health-system managers should shape symptom language, referral thresholds, treatment options, consent procedures, review intervals, accountability structures and locally meaningful outcomes. Delphi or other consensus methods can refine minimum data elements, first-line treatment boundaries, referral principles and review expectations without imposing a universal protocol. Pragmatic implementation studies should then test feasibility, acceptability, fidelity, cost, equity and effects on recognition, review, referral and continuity, while monitoring overdiagnosis, excessive hormonal suppression, delayed alternative diagnosis, referral bottlenecks and inequitable access.

Success should be judged by earlier identification of clinically significant symptoms, better documentation of fertility intentions, fewer repeated unreviewed treatments, more appropriate referral, higher review completion and improved patient-reported function and validation. Failure would include missed alternative diagnoses, indefinite empirical care, inappropriate referral, specialist congestion, poor follow-up or widening inequities. The framework is therefore testable and should be confirmed, modified or rejected on empirical evidence.

This article makes a bounded claim: endometriosis care in low-resource settings requires explicit pathway logic that goes beyond awareness campaigns or expanded access to laparoscopy alone. Specialist imaging, surgery and advanced fertility care remain essential for selected patients, but waiting for tertiary confirmation before meaningful action risks untreated pain, repeated low-value care, delayed fertility evaluation and fragmented continuity. The 5R Framework therefore proposes that endometriosis-compatible symptoms should trigger recognition, safe rule-out, timely relief, need-based referral and planned review. Its value will ultimately depend on whether context-specific adaptation and testing improve timely, safe and equitable care without weakening diagnostic caution.

## Data Availability

The original contributions presented in the study are included in the article/Supplementary Material, further inquiries can be directed to the corresponding author/s.
